# Identification of Freezing-Responsive microRNAs and Their Targets in Chinese Jujube by Small RNA and Degradome Sequencing

**DOI:** 10.3390/ijms27177912

**Published:** 2026-09-04

**Authors:** Luhe Zhang, Mei Liu, Xiaoqin Duan, Chengying Jiang, Tong Zhao, Junying Zhao

**Affiliations:** 1 Gansu Academy of Forestry Sciences, Lanzhou 730020, China; zhangluhe@126.com (L.Z.); jcytxb@126.com (C.J.); zhaot3081@126.com (T.Z.); 2 Institute of Fruit and Floriculture Research, Gansu Academy of Agricultural Sciences, Lanzhou 730070, China; 3 Agricultural College, Gansu Agricultural University, Lanzhou 730070, China; liumei0460@163.com (M.L.); dxq2755545382@163.com (X.D.); 4 National Forestry and Grassland Administration Key Laboratory of Olive, Lanzhou 730020, China

**Keywords:** microRNA, deep sequencing, jujube, cold stress, degradome sequencing

## Abstract

The jujube tree fruit remains a primary fruit in northern China, yet its geographical distribution and yield are significantly constrained by freezing stress during winter. Numerous studies have highlighted the pivotal regulatory function of microRNAs (miRNAs) in plant responses to low-temperature stress. Nevertheless, the specific miRNAs involved in the response to low temperatures and their associated gene networks in *Ziziphus jujuba* Mill are not well understood. In this investigation, we utilized high-throughput sequencing to analyze small RNA libraries from branches subjected to temperatures of 4 °C and −30 °C. Our analysis identified a total of 342 miRNAs, comprising 123 known miRNAs and 219 novel miRNAs. The differential expression analysis revealed that under low-temperature conditions, 177 miRNAs underwent significant changes. Among them, specific upregulation of miR319 in the less cold-resistant variety and miR6483 in sensitive variety was observed. By employing degradome sequencing, we identified a total of 1551 target genes corresponding to 3059 unique miRNA target interaction pairs involving 299 miRNAs. Functional analysis using Gene Ontology (GO) and Kyoto Encyclopedia of Genes and Genomes (KEGG) pathways indicated that these target genes are primarily associated with transcriptional regulation, metabolic pathways, and genetic information processing. Through a comprehensive analysis, we pinpointed 11 genes corresponding to 9 miRNAs that are implicated in jujube tree cold stress, and 7 target genes of 7 miRNAs were confirmed by 5′-RACE analysis. These miRNAs are likely to exert crucial regulatory functions in the context of jujube tree cold stress. This study is the first to systematically identify miRNAs and their target genes in the response of *Ziziphus jujuba* Mill to low-temperature stress, which provides important resources for in-depth analysis of the molecular mechanism of jujube tree cold resistance and for cold-resistant breeding.

## 1. Introduction

Recent studies have uncovered that small RNAs have a broad impact on post-transcriptional gene expression regulation, significantly altering our comprehension of gene regulatory mechanisms in development and diverse biological processes, thereby representing a pivotal tier of gene expression control [1,2]. The pathways and key enzyme components are well described and are highly conserved in plants and animals [3]. miRNA is an endogenous, single-stranded, non-coding small RNA (sRNA) approximately 18–24 nucleotides in length. It silences gene expression by either cleaving the mRNA of target genes or inhibiting their translation. This mechanism is crucial for regulating plant growth, development, and environmental adaptation, and it plays a particularly significant role in mediating plant responses to both biotic and abiotic stresses [4]. In recent years, numerous miRNAs have been identified in plant genomes [5], suggesting that the identification of their target RNAs is essential for the functional analysis of miRNA.

Low temperature is a prevalent abiotic stressor that can inflict damage on plant tissues and hinder growth, thereby significantly impairing development, diminishing crop productivity, and constraining the natural geographical distribution and cultivation range of species [6,7,8]. Cold stress can be categorized based on temperature variations into cold damage (≤20 °C) and freeze damage (≤0 °C), both of which are critical environmental factors influencing the distribution patterns, growth rhythms, and yield formation of plants. It is important to note that while temperatures below 20 °C constitute cold stress for cold-sensitive species, many cold-tolerant plants have optimal growth temperatures near or below this range and only experience stress when temperatures drop further toward freezing or below. Cold-tolerant plants respond to and adapt to cold stress through various biochemical changes, including the accumulation of metabolites [9,10], the synthesis of unsaturated fatty acids [11], the production of antifreeze proteins [12], and the increase of heat shock proteins [13]. In addition to the classic transcriptional regulatory mechanisms, miRNAs also play significant roles as regulatory factors in the gene regulatory network of plant cold responses. Various miRNAs involved in the cold stress responses have been identified successively.

At present, many miRNAs responding to cold stress have been identified in various plants, such as wheat [14], corn [15], rice [16,17], *Arabidopsis thaliana* [18], poplar (*Populus tomentosa*) [19], *Camellia sinensis* [20], tomato [21], etc. The first plant defense miRNA discovered was miR393 in *Arabidopsis* [22,23], which could increase the amount of cold-induced proteins by depressing its target gene (ubiquitin E3 ligase) [24,25]. A recent study demonstrated that miR168, miR397, miR164, and miR1029 exhibit differential expression in response to cold stress in the wheat genotype C-306 [26]. In rice, miR156 enhances tolerance to low temperatures by targeting and regulating transcription factor genes, such as SQUAMOSA promoter binding protein-like (SPL) [27]. Additionally, in maize, miR166c-5p is upregulated following cold stress exposure [15]. In transgenic tomatoes, the expression of the miR399d gene is moderately upregulated in response to both salt and cold stress [28]. Collectively, these findings underscore the critical role of miRNAs in cold stress responses, highlighting the practical significance of identifying miRNAs associated with cold stress in *Ziziphus jujuba* Mill.

The jujube tree (*Ziziphus jujuba* Mill.) is a deciduous fruit tree that is native to China, where it has been cultivated for a period exceeding 3000 years. Its distribution has now spread across the globe, and it has gained significant ecological, economic, and medicinal value [29]. In recent years, there has been a notable increase in the area dedicated to the cultivation of *Ziziphus jujuba* Mill, accompanied by a concomitant expansion in their distribution range. However, in the northern regions, winter temperatures are comparatively low, and extreme cold is frequently experienced. Frost damage to *Ziziphus jujuba* Mill is a common occurrence, particularly among juvenile trees and seedlings, which are susceptible to severe freezing. The issue of low-temperature damage during the winter and spring months has emerged as a significant challenge for jujube production in northern regions, representing a substantial threat to the advancement of the jujube industry. The suitable growth temperature for *Ziziphus jujuba* Mill is 18 to 30 °C, and the optimal temperature for the flowering period is 23 to 26 °C. During the dormancy period, if the temperature is continuously lower than −18 °C, it is prone to cause frost damage. After the buds start to sprout, the cold resistance significantly decreases, and low temperatures of 0 °C and below are likely to damage the tender shoots, flowers and fruits [30,31]. For instance, in the majority of regions within Xinjiang, a range of measures must be implemented to ensure the protection against low temperatures during the winter months. This has a significant impact on the production costs of jujubes, hindering both the financial gain of farmers and the long-term growth of the jujube industry as a whole. Consequently, the most efficacious method of safeguarding *Ziziphus jujuba* Mill from the deleterious effects of low-temperature climates is to breed varieties that possess robust cold resistance. The small size of jujube tree flowers makes manual removal of the stamens difficult, the fruit setting rate is low, and there is a severe embryo abortion phenomenon, which makes hybrid breeding of *Ziziphus jujuba* Mill very difficult. Consequently, biotechnology breeding represents a pivotal future direction for the cultivation of *Ziziphus jujuba* Mill.

Currently, there is a limited number of studies identifying the cold resistance of jujube tree germplasm varieties, and research on the molecular mechanisms underlying their cold resistance is even more sparse. Consequently, there is an insufficient theoretical foundation for employing biotechnology to breed and cultivate jujube tree varieties with enhanced cold resistance. While some investigations have addressed jujube tree diseases [32], drought resistance [33], and miRNA responses under salt stress [34], there is a notable absence of reports concerning the regulation of miRNA in *Ziziphus jujuba* Mill in response to low-temperature stress. Therefore, this study selected the cold-resistant jujube varieties ‘Linzexiaozao’ and the less cold-resistant cultivar ‘Jinchang No.1’ as research materials. High-throughput sequencing technology was utilized to identify miRNAs that respond to low-temperature stress in *Ziziphus jujuba* Mill. A total of 342 miRNAs were identified in the small RNA library. The targets of these miRNAs were predicted and validated through degradome sequencing, and their biological functions were subsequently annotated. The miRNAs and their target genes identified in this study will provide a foundation for a deeper understanding of the molecular mechanisms by which *Ziziphus jujuba* Mill respond to low-temperature signal transduction.

## 2. Results

### 2.1. MiRNA Sequencing Data Analysis

**Figure 1:**
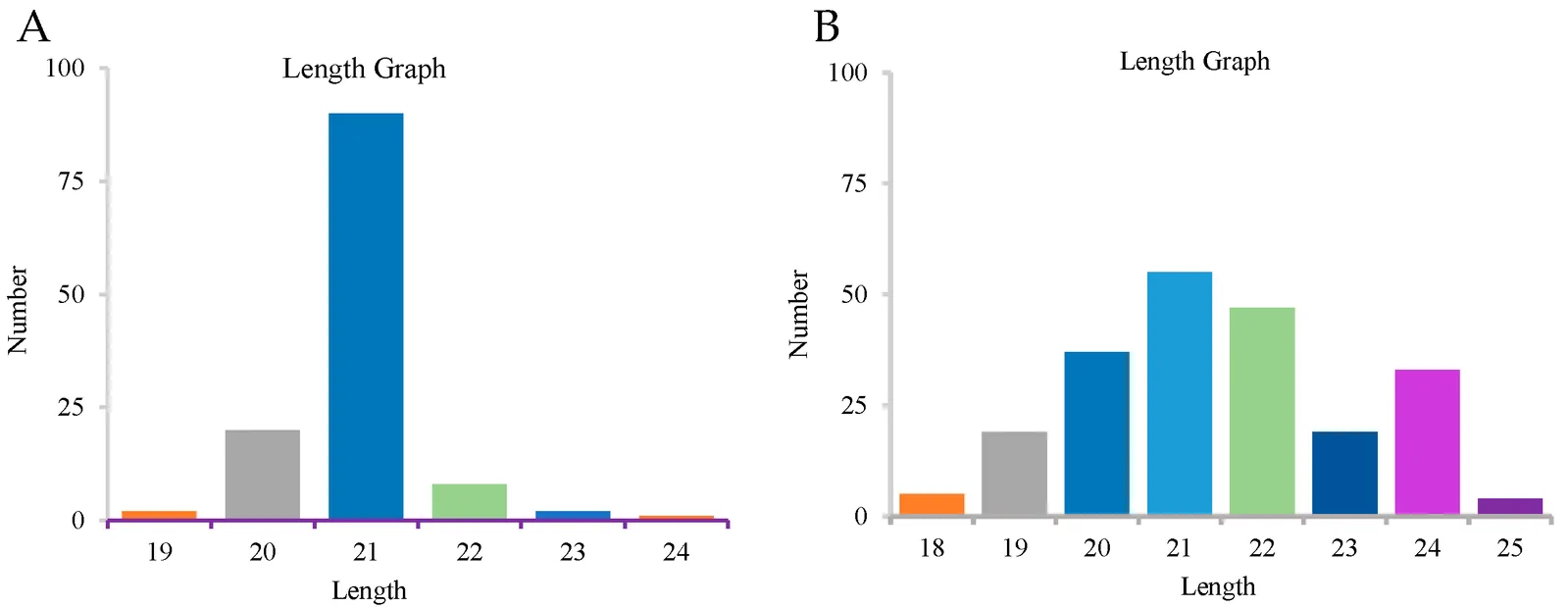
Length distribution of small RNAs. (**A**) Length distribution of known miRNAs. (**B**) Length distribution of novel miRNAs.

**Figure 2:**
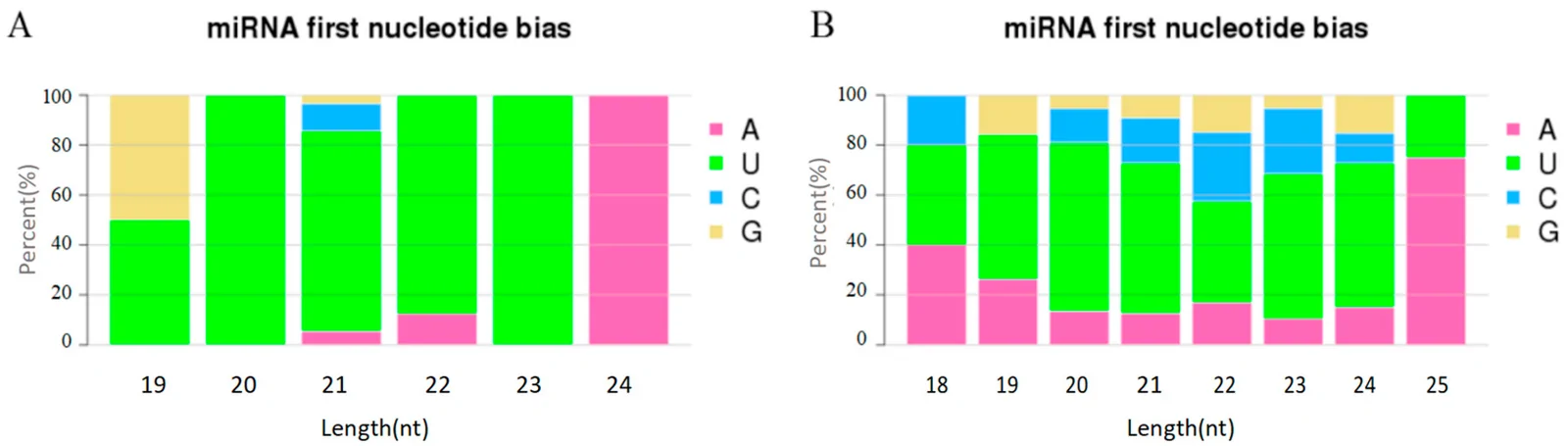
Nucleotide bias of miRNA first nucleotide bias analysis. (**A**) First nucleotide bias of known miRNAs. (**B**) First nucleotide bias of novel miRNAs.

**Figure 3:**
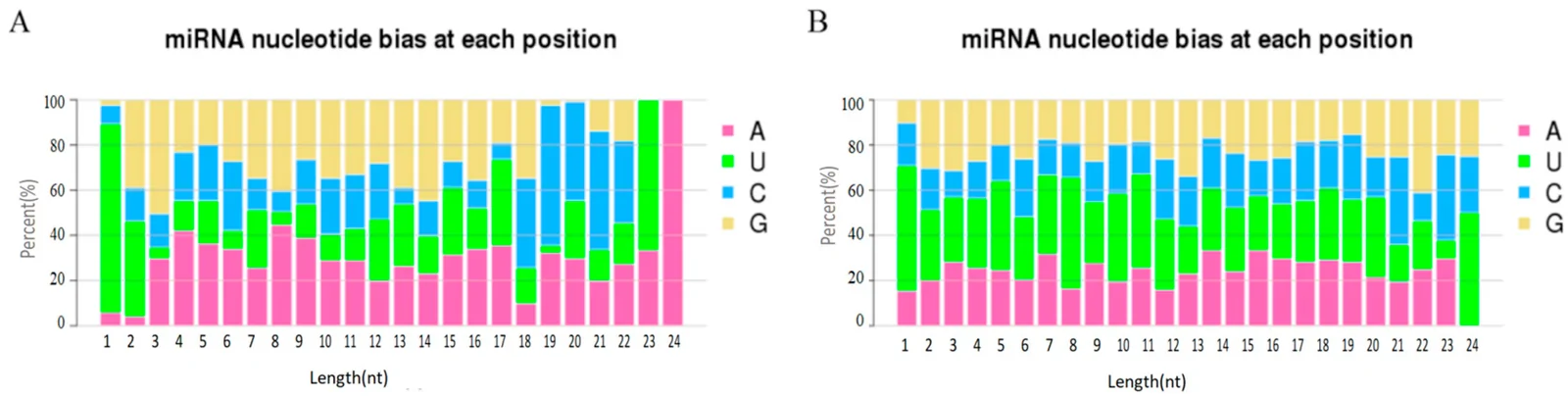
Nucleotide bias at each position of miRNAs. (**A**) The nucleotide bias of known miRNAs. (**B**) The nucleotide bias of the novel miRNAs.

This study utilized the cold-resistant variety ‘Linzexiaozao’ and the less cold-resistant variety ‘Jinchang No.1’ as research materials. A control temperature of 4 °C was established, while the low-temperature treatment was set at −30 °C. Small molecule RNA libraries were constructed for both the control and low-temperature treatment samples and sequenced. A total of 11,880,054 to 25,731,472 raw sequencing reads were obtained from the control and low-temperature treatment samples (Table 1). Following the removal of low-quality sequences, sequences with a length shorter than 18 nt or longer than 30 nt, and Containing ‘N’ reads, 9,599,285 to 19,680,747 clean reads were retained from both sample sets (Table 1). BLAST (Version 2.16.0) analysis further annotated the clean reads as rRNA, snRNA, snoRNAs, and tRNAs. Unannotated reads were aligned to the Jujube tree genome, revealing alignment rates for the 12 samples that ranged from 53.77% to 72.33% (Table 2). Length distribution analysis indicated that the majority of annotated reads were between 20 and 24 nt, aligning with the typical characteristics of Dicer enzyme cleavage and corroborating findings from other plant studies (Figure 1). Base preference analysis of the obtained known and newly predicted miRNAs revealed that U had a higher frequency of occurrence in the 5′ end of miRNAs (Figure 2 and Figure 3).

### 2.2. Identification of Known and New miRNAs in Ziziphus jujuba Mill

To identify the known miRNAs in *Ziziphus jujuba* Mill, unannotated small RNA sequences were compared with the miRBase database (v22.0). A total of 342 miRNAs were identified, comprising 123 known miRNAs and 219 newly predicted miRNAs (Table 3; Supplementary Materials S1 (Table S1) and S2), which are distributed across 61 miRNA families. The number of members within each family varies significantly; the MIR156 family contains the most members, totaling 21. The second largest family, MIR171_1, consists of 13 members. Notably, one family has 33 members, accounting for 54.10% of the total (Supplementary Material S3 (Table S3)). Expression levels differ among families, and even among members within the same family, expression levels can vary.

### 2.3. Expression Patterns of miRNAs in *Ziziphus jujuba* Mill

**Figure 4:**
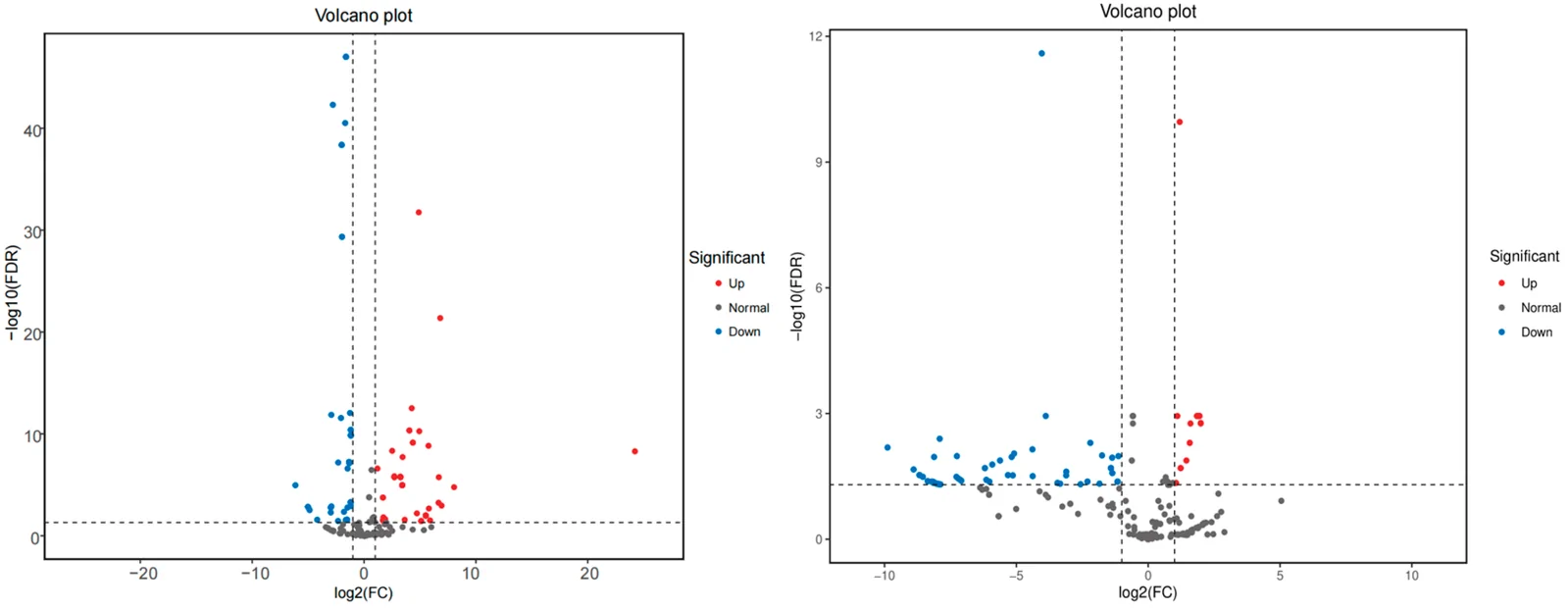
Volcano plot of differentially expressed miRNAs in response to freezing treatment in *Ziziphus jujuba* Mill. Note: left: JCK vs. JT. right: LCK vs. LT. Differential expression was analyzed using DESeq2 with |log_2_(fold change)| ≥ 1.00 and FDR ≤ 0.05 as the threshold. log_2_ (fold change) is defined as log_2_(treated/control), where ‘treated’ indicates the cold-treated group and ‘control’ indicates the corresponding control group.

**Figure 5:**
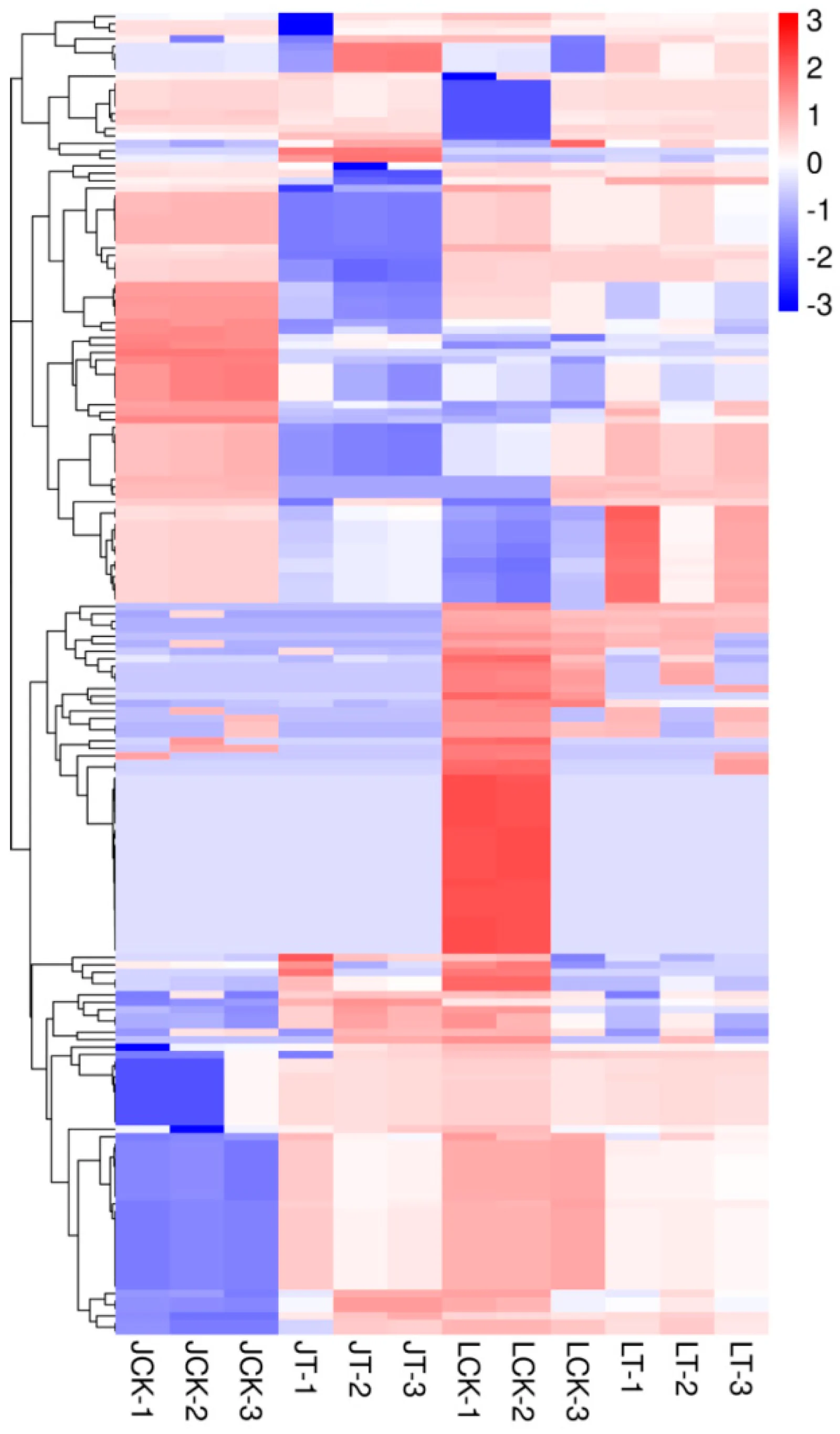
Clustering diagram of differentially expressed miRNAs. Note: The above figure is a clustering diagram of differentially expressed miRNAs. The columns represent different samples, and the rows represent different miRNAs. The clustering is based on the log10(TPM + 1 × 10^−6^) value. Red indicates high-expression miRNAs, and blue indicates low-expression miRNAs. CK, JT, LCK, LT are the same as above.

To identify the miRNAs associated with cold resistance in *Ziziphus jujuba* Mill, this study employed DESeq2 to perform differential expression analysis using raw integer counts as input. A total of 177 differentially expressed miRNAs (DEMs) were identified as the union of the DEMs detected in the two cultivar-specific comparisons, comprising 57 known miRNAs and 120 novel miRNAs (Figure 4 and Figure 5; Supplementary Material S4 (Table S4)). In the JCK and JT groups, 112 miRNAs exhibited differential expression in jujube branches, with 61 miRNAs upregulated and 51 miRNAs downregulated (Supplementary Material S5 (Table S5)). In the LCK and LT groups, 102 miRNAs were differentially expressed, with 22 miRNAs, including miR319 (in the miR319 family) and miR159, upregulated and 80 miRNAs downregulated (Table 4; Supplementary Material S6 (Table S6)).

### 2.4. Identify miRNA Targets Through Degradation Group Analysis

To investigate the functions of miRNAs in response to low-temperature stress in *Ziziphus jujuba*, two mixed degradome libraries (JC1 and LZXZ) were constructed, of which one mixed degradome library (JC1) was constructed by pooling equal amounts of total RNA from JCK and JT samples; another mixed degradome library (LZXZ) was constructed by pooling equal amounts of total RNA from LCK and LT samples. The objective was to identify mRNA cleavage sites mediated by miRNAs at the whole transcriptome level. Sequencing of the mixed degradation group generated a total of 20,035,872 raw reads. Following adapter removal and length filtering, 8,653,275 unique alignable reads were obtained, with 42.39% of these unique sequences successfully aligned to the jujube genome. The transcript coverage ranged from 69.04% to 70.90% (Table 5). All mapped reads were subsequently analyzed using the CleaveLand v4.0 program, resulting in the identification of 3059 unique miRNA target gene interaction pairs involving 299 miRNAs in *Ziziphus jujuba* trees, which correspond to 1551 unique target genes (Supplementary Material S7 (Table S7)).

Among these miRNAs, the recently identified novel_miR189 from *Ziziphus jujuba* Mill exhibited the highest number of predicted target genes, totaling 447. These target genes are primarily enriched in biological processes related to cold stress response, reactive oxygen species clearance, osmotic stress protection, protein folding stability, and signal transduction. Notably, its target genes encompass antioxidant-related genes, including glutathione S-transferase, ascorbate peroxidase, and thioredoxin, along with stress-protective genes such as osmotic proteins and dehydration proteins.

### 2.5. GO and KEGG Analysis of miRNA Target Genes in Ziziphus jujuba Mill

**Figure 6:**
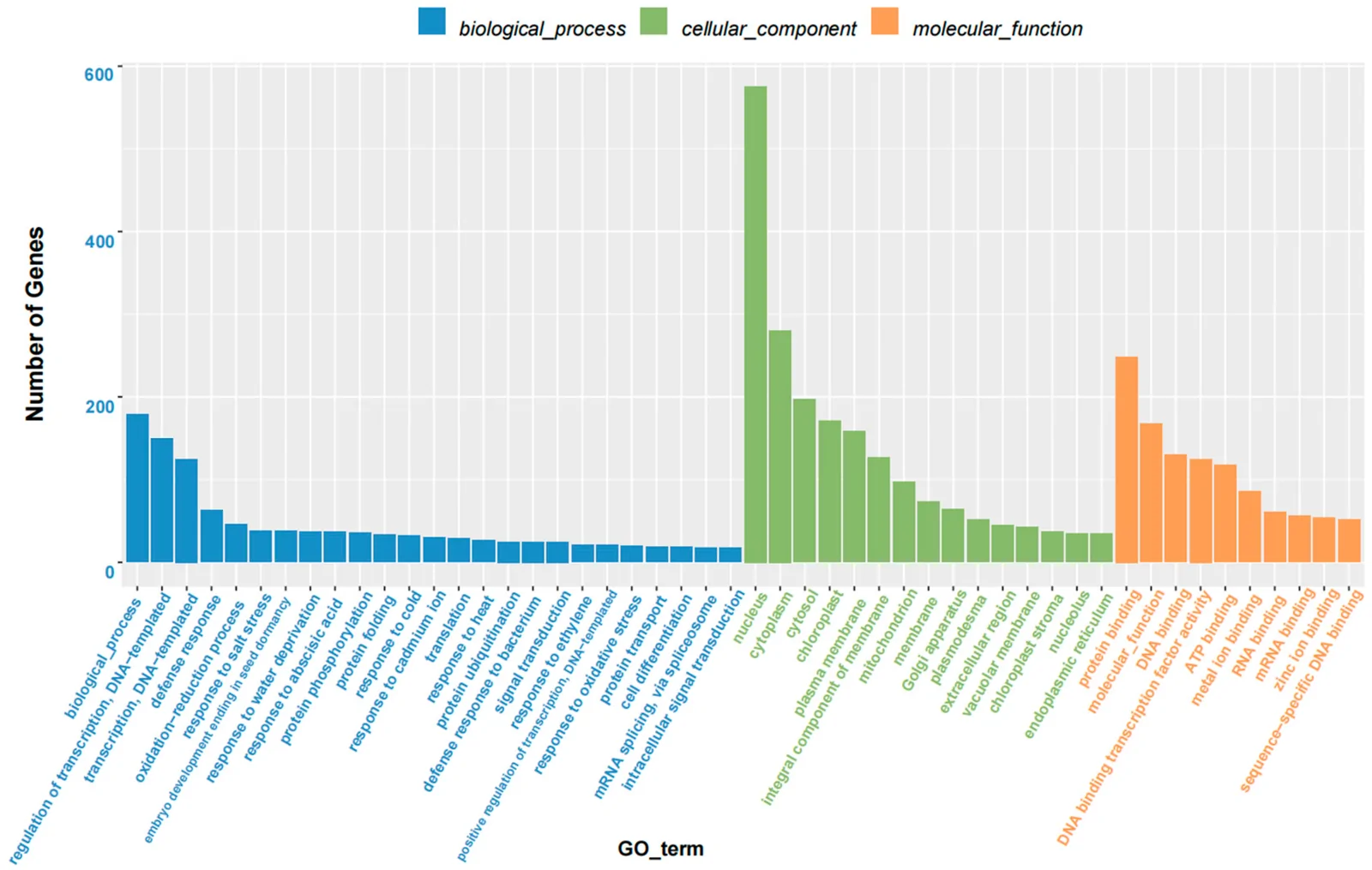
GO annotation of target genes for degradation sequencing.

**Figure 7:**
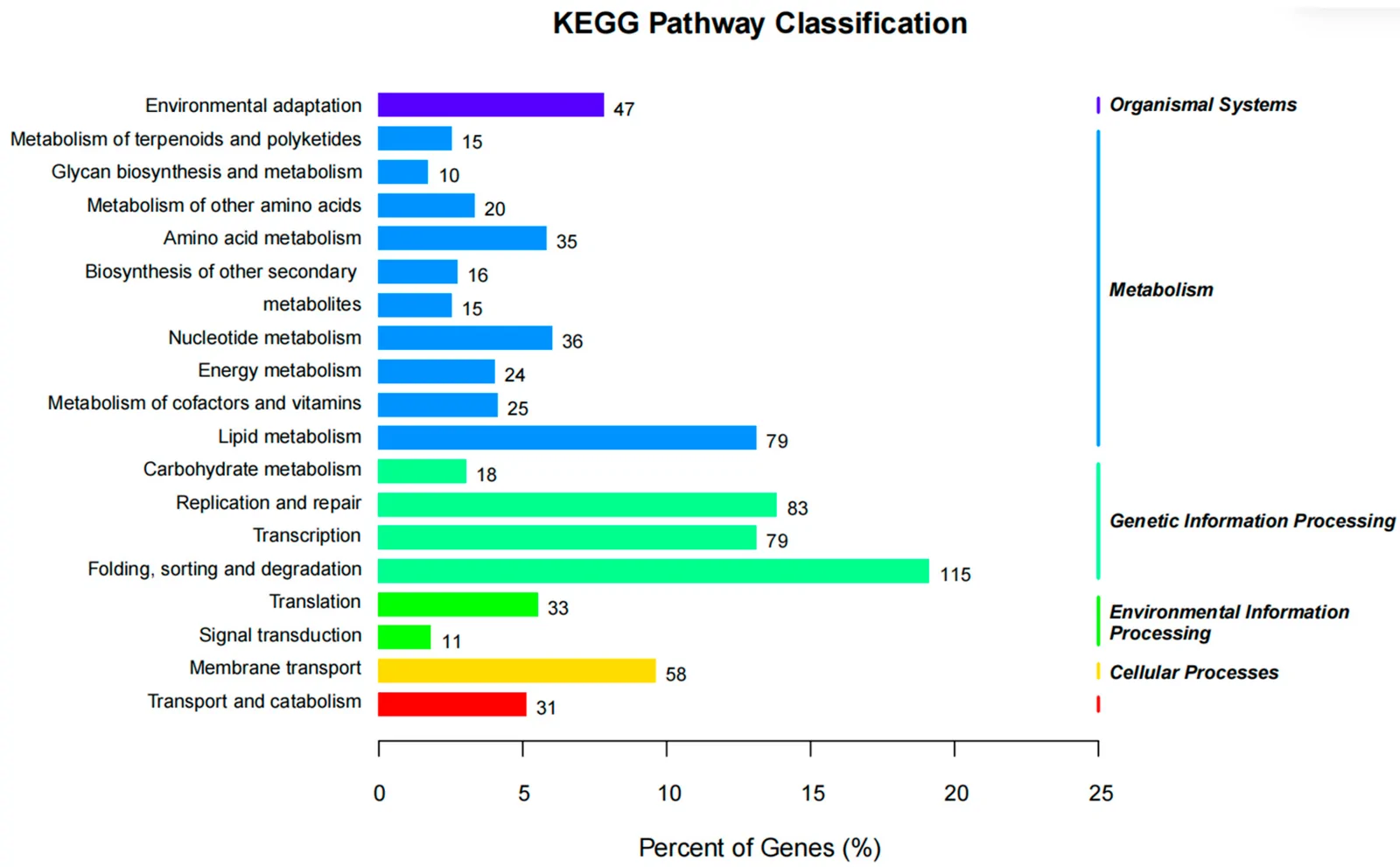
KEGG pathway analysis of the target genes.

GO analysis indicated that the functions of all target genes could be categorized into 25 biological processes, 15 cellular components, and 10 molecular functions (Figure 6). Within the biological processes, the target genes were predominantly enriched in areas such as regulation of biological processes, DNA-templated transcription, and transcriptional regulation. The cellular components were significantly enriched in the nucleus, cytoplasm, and cytoplasmic matrix. In terms of molecular functions, the target genes concentrated on essential activities such as protein binding and DNA binding, The KEGG analysis found that these target genes were mainly involved in 113 pathways such as environmental adaptation, metabolic processes, transcription, folding, sorting and degradation, among which most target genes were enriched in pathways related to metabolism and genetic information processing (Figure 7).

### 2.6. Identification of miRNAs and Their Target Genes Responding to Low-Temperature Stress

**Figure 8:**
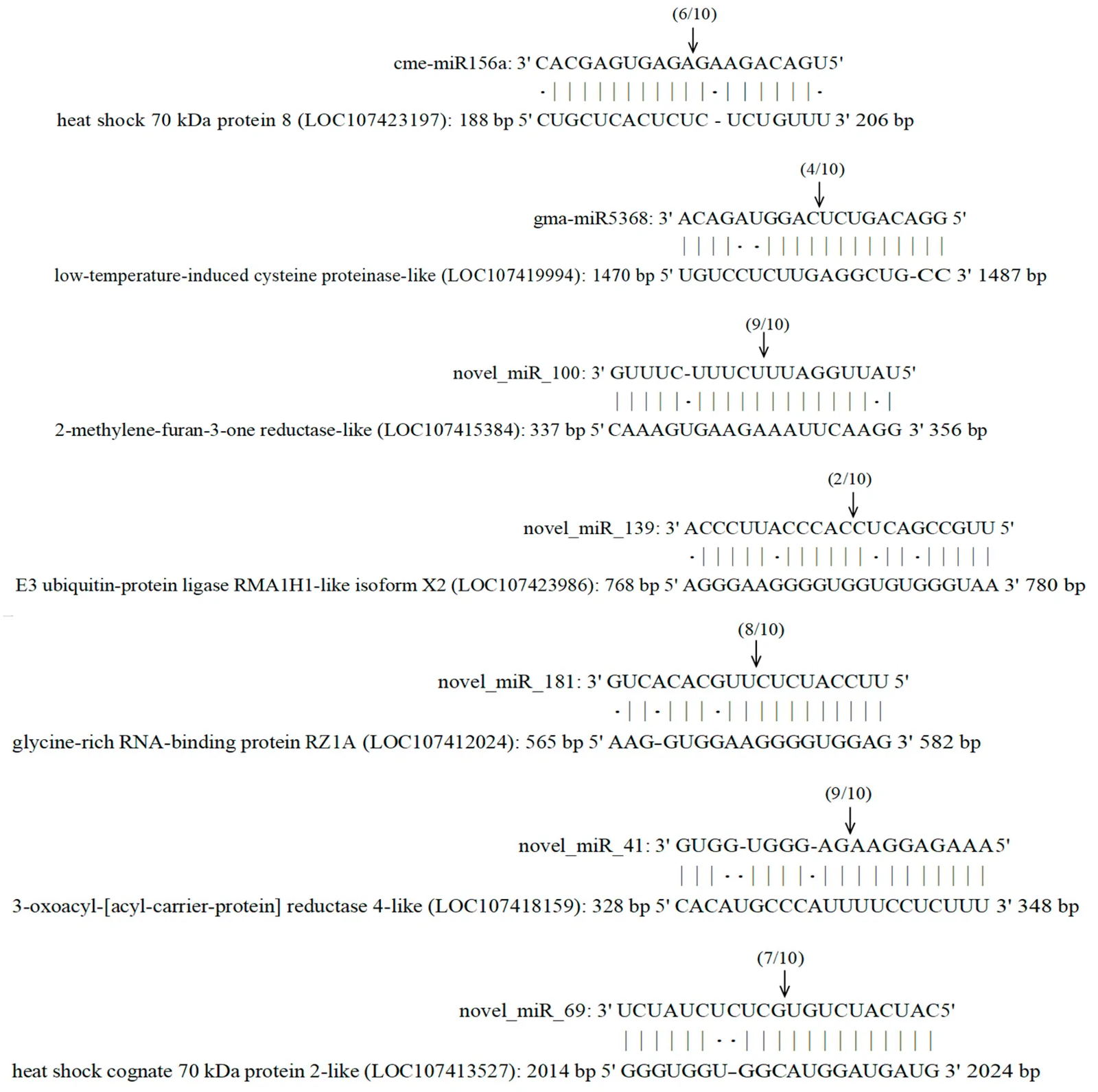
RLM-5′ RACE verification of target mRNA cleavage sites of cold stress-related miRNAs. Fraction within parentheses means the proportions of 5′ RACE clones showing these cleavage sites out of all sequenced clones.

Based on the Gene Ontology annotation of miRNA target genes and literature review, we identified a total of 11 target genes of 9 miRNAs that are involved in the cold response of *Ziziphus jujuba* Mill (Table 6). To validate the target genes of cold stress-related miRNAs in vivo of Chinese jujube, we performed RLM-5′ RACE assay to amplify cleavage position of potential miRNA target gene transcripts, and found that 7 target genes of 7 miRNAs were cleaved at the middle of the miRNA binding regions of mRNA fragment (Figure 8), which including heat shock 70 kDa protein, low-temperature-induced cysteine proteinase-like, 2-methylene-furan-3-one reductase-like, bifunctional hydroxyethylthiazole kinase/thiamine-phosphate pyrophosphorylase, E3 ubiquitin-protein ligase RMA1H1-like isoform X2, glycine-rich RNA-binding protein RZ1A, 3-oxoacyl-[acyl-carrier-protein] reductase 4-like, 3-ketoacyl-CoA thiolase 2, peroxisoma, heat shock cognate 70 kDa protein 2-like. Our results suggested that those genes were targeted by cold-related miRNAs in Chinese jujube.

### 2.7. Detection of the Expression of miRNA and Their Targets by qRT-PCR

**Figure 9:**
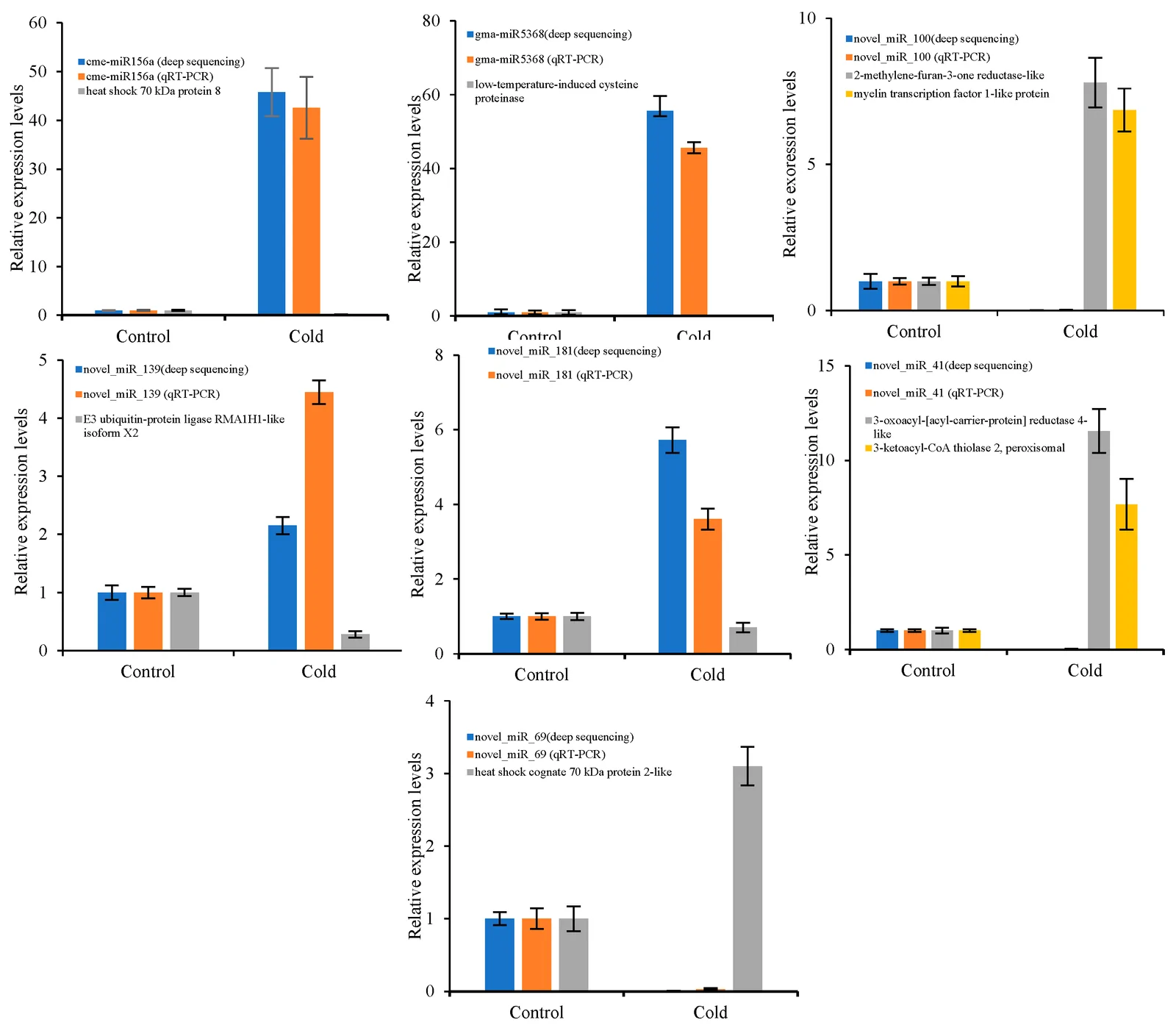
RT-qPCR analysis of the expression levels of potential chilling-responsive miRNAs and their targets.

To validate the sequencing results, we performed additional verification of the 7 miRNAs associated with the low-temperature response in *Ziziphus jujuba* Mill, along with their target genes, using qRT-PCR. The findings indicated that the all of the differentially expressed miRNAs displayed expression patterns consistent with those identified in the sequencing data, while the expression alterations of their target genes were negatively correlated with the differentially expressed miRNAs (Figure 9). It indicates that the results from sequencing are reliable and may provide an accurate indication of the level of miRNA expression in Jujube tree.

## 3. Discussion

MiRNAs play key roles in most biological processes in animals and plants [35,36]. To date, high-throughput sequencing techniques have revealed a substantial number of miRNAs associated with various biological pathways in multiple species. Nevertheless, the systematic identification of miRNAs in *Ziziphus jujuba* Mill remains limited, particularly concerning those that respond to cold stress. This study employs a combined analysis of small RNA sequencing and degradome sequencing to systematically identify miRNAs and their target genes that respond to cold stress in *Ziziphus jujuba* Mill. The aim is to elucidate the regulatory mechanisms of miRNAs in the cold adaptation process of these trees.

The small RNA analysis of *Ziziphus jujuba* Mill identified 123 known miRNAs and 219 novel miRNAs (Table 3), with lengths ranging from 18 to 25 nt. Notably, sequences of 21 nt constituted the highest proportion (Figure 1). This length distribution of miRNAs aligns with observations in other plant species [37,38]. The expression difference analysis revealed 177 differentially expressed miRNAs (DEMs) (Table 4). Compared to the control group, the majority of DEMs were significantly downregulated under low-temperature treatment (Figure 4). This finding is consistent with the results reported by Kou et al. [39] in potatoes. Furthermore, we observed that the number of upregulated miRNAs in cold-resistant varieties was considerably lower than that in sensitive varieties, corroborating previous reports that cold-resistant varieties exhibit smaller changes in miRNA expression under cold stress [40]. Further analysis indicates that miR319 was upregulated under low temperature in the less cold-resistant variety ‘Jinchang No.1’ (LCK vs. LT comparison; Supplementary Material Table S6), while its target, TCP transcription factor, typically inhibits the expression of cold-responsive genes. This finding is consistent with results in rice, where overexpression of miR319 enhances cold resistance [16]. Additionally, miR156 exhibited differential expression under low temperature, and prior studies have demonstrated that miR156 is involved in cold signal transduction by targeting SPL transcription factors [27]. This further substantiates the potential regulatory role of miR156 in the cold response of *Ziziphus jujuba* Mill.

To verify the cleavage events of miRNAs on target genes, this study employed degradation sequencing. A total of 1551 target genes corresponding to 3059 unique miRNA target interaction pairs involving 299 miRNAs were identified (Table 5; Supplementary Material S7 (Table S7)). Notably, the novel miRNA, novel_miR189, targets up to 447 genes (Supplementary Material S7 (Table S7)), making it one of the miRNAs with the highest number of target genes among known plant cold response miRNAs. These target genes are extensively involved in antioxidant defense, osmotic regulation, protein homeostasis, and the modulation of various transcription factors. Furthermore, this miRNA can interact with various transcription factors (Figure 6 and Figure 7), such as WRKY, bHLH, bZIP, NAC, ERF, and CBL-interacting protein kinases, thereby participating in the cold signal transduction and transcriptional regulatory network. It also regulates hormone signals, including auxin and abscisic acid, as well as fundamental life processes such as RNA processing, translation, and ribosome assembly, thus playing a significant and multifaceted regulatory role in the cold stress response of the jujube tree. Similar multi-target regulatory phenomena have been documented in the drought stress response of poplar; however, the scale of such phenomena is considerably smaller than that observed in this study [5].

miRNA typically regulates gene expression through two mechanisms: it either mediates the cleavage of target mRNA or inhibits its translation [41,42]. In plants, the cleavage of target mRNA appears to be the predominant mode of gene regulation by miRNA [43]. To elucidate the nature of miRNA target genes associated with cold stress in *Ziziphus jujuba* Mill and their regulatory effects, we identified seven cold stress-related target genes using RLM-5′ RACE detection. The results indicated that 7 miRNAs corresponded to 7 target genes, with specific cleavage occurring within their binding regions. Functional classification of these target genes revealed their significant involvement in multiple key physiological processes related to the plant’s cold stress response. Notably, Hsp70 s (heat shock proteins 70 s) represent a class of molecular chaperone proteins that are highly conserved and widely distributed across microorganisms, plants, and humans. The hsp70 gene has been implicated in cold stress responses in various organisms, including *Arabidopsis thaliana* [44], pineapple [45], maize [46], and peas [47]. Additionally, another study demonstrated that the interaction between miRNAs and cysteine protease genes plays a role in regulating the plant’s response to low temperatures [48]. The E3 ubiquitin-protein ligase may play a role in regulating plant cold responses by influencing the ubiquitin–proteasome pathway. Research indicates that re-linking the E3 ligase can enhance cold tolerance in corn [49]. The E3 ubiquitin ligase OsPUB9 negatively regulates cold tolerance in rice [50]; additionally, the glycine-rich RNA-binding protein (RZ1A) is recognized as a regulatory factor in cold responses. In *Arabidopsis thaliana*, *AtRZ-1a* expression is significantly induced under low-temperature stress [51]. Other target genes, including 3-oxoacyl-ACP reductase, 3-ketoacyl-CoA thiolase, and peroxidase-related protein, are involved in fatty acid synthesis, fatty acid β-oxidation, and the removal of reactive oxygen species. These processes are crucial for energy metabolism and membrane protection during plant cold adaptation [52,53,54]. Collectively, these findings suggest that miRNAs and their targets likely participate in regulating cold stress in *Ziziphus jujuba* Mill. This study offers important experimental evidence for understanding the post-transcriptional regulatory mechanisms mediated by miRNAs in the cold adaptation of *Ziziphus jujuba* Mill.

In conclusion, this study represents the first comprehensive identification of conserved and specific miRNAs associated with freezing stress in *Ziziphus jujuba* Mill at the whole-genome level, along with their corresponding target genes. This foundational work will facilitate further investigations into the regulatory mechanisms governing miRNAs linked to cold tolerance in *Ziziphus jujuba* Mill. Nonetheless, additional experiments are necessary to ascertain whether these differentially expressed miRNAs directly influence the regulation of cold tolerance in *Ziziphus jujuba* Mill.

## 4. Materials and Methods

### 4.1. Plant Materials

The study utilized the cold-resistant cultivar ‘Linzexiaozao’ (LZXZ) and the less cold-resistant cultivar ‘Jinchang No.1’ (JCK) of Chinese jujube (*Ziziphus jujuba* Mill.) as experimental subjects. Based on previous evaluations of their freezing tolerance under field conditions [30], ‘Linzexiaozao’ can tolerate temperatures as low as −35 °C with less than 30% electrolyte leakage, whereas ‘Jinchang No.1’ shows severe damage (>70% electrolyte leakage) at −30 °C, making it a sensitive control for cold tolerance studies. One-year-old branches (approximately 1.0–1.5 cm diameter, 10 cm in length) from healthy trees of each cultivar were collected; branches were processed within 20 min of cutting from the tree. Sections were thoroughly washed with tap water, rinsed thrice with deionized water, and subsequently enclosed in sealed bags to prevent desiccation during treatment.

Temperature treatments were performed using a programmable low-temperature incubator (LRH-250CB, Shanghai Yiheng Scientific Instruments, Shanghai, China). For the control group, branches were maintained at 4 °C for 12 h. For the low-temperature treatment, branches were cooled from 4 °C to −30 °C, and held at −30 °C for 12 h. Following temperature treatment, branch tissues were immediately harvested, snap-frozen in liquid nitrogen, and stored at −80 °C for RNA extraction.

### 4.2. Small RNA Library Construction and Sequencing

Total RNA was extracted from jujube tree branch samples stored at 4 °C and −30 °C following the manufacturer’s instructions employing the Total RNA Extraction Kit from Tiangen Biotech, Beijing, China. The RNA samples underwent analysis for purity, concentration, and integrity utilizing sophisticated molecular biology equipment. Subsequently, small RNAs ranging from 18 to 30 nucleotides were isolated via gel electrophoresis, purified, and prepared for sequencing. Lastly, a small RNA (sRNA) library was generated, and Illumina SE50 sequencing system (Illumina, San Diego, CA, USA) was utilized for single-end sequencing.

### 4.3. Identification of Known and Novel miRNAs

The raw RNA sequencing data obtained from high-throughput sequencing underwent preprocessing to eliminate low-quality tags and generate small RNA (sRNA) tags. Subsequently, additional filtering was performed using a Perl script to remove adapter sequences, sequences containing poly-N, reads shorter than 18 nucleotides or longer than 30 nucleotides, and low-quality reads. The Bowtie v1.1.1 software [55] was employed on a high-performance computing platform to compare the high-quality clean reads, ranging from 18 to 30 nucleotides, against the Silva, GtRNAdb, Rfam, and Repbase databases, thereby excluding non-coding RNAs such as rRNA, tRNA, snRNA, and snoRNA. The remaining sequences were then compared with the miRBase (v22) database; those that matched were classified as known miRNAs, while unannotated sequences were predicted as novel miRNAs using the miRDeep2 v2.0.5 software and modified the length of miRNA precursor sequence from 100 nt to 250 nt.

### 4.4. Differential Expression Analysis

To identify differentially expressed miRNAs between the cold-resistant cultivar ‘Linzexiaozao’ and the less cold-resistant cultivar ‘Jinchang No.1’ under low-temperature stress, we first obtained the raw read counts for each miRNA from the small RNA sequencing data. DESeq 1.39.0 software [56] was then applied to perform differential expression analysis using the raw integer counts as input, following the negative binomial distribution model implemented in the package. miRNAs with |log2(fold change)| ≥ 1.00 and FDR ≤ 0.05 were considered differentially expressed. The fold change was calculated as the expression level ratio of the cold-treated group to the control group, i.e., log2(fold change) = log2(treated/control). For data visualization purposes only, miRNA expression levels were normalized to transcripts per million (TPM) using the formula: TPM = read count × 1,000,000/mapped reads, to represent relative abundance across libraries.

### 4.5. Prediction of miRNA Targets and Functional Annotation

To identify the true target genes of miRNAs that respond to low-temperature stress, this study performed degradome sequencing on samples from both the control group (JC1) and the low-temperature treatment group (LZXZ). Degradome library construction was performed using the Illumina TruSeq Small RNA Sample Preparation Kit following the manufacturer’s protocol. Libraries were sequenced on an Illumina HiSeq 2500 platform. The CleaveLand v4.0 program [57] was employed for quality control of the raw data, and redundancies were eliminated. Subsequently, Oligomap was utilized to align the comparable sequences with the cDNA database, resulting in the generation of a degradation group density file [58]. For each matching site, a 26-nt sequence, comprising 13 nt upstream and 13 nt downstream, was extracted. The score was calculated using EMBOSS Needle and the small RNA library comparison, following the plant miRNA/target pair standard [59]. GSTAr was then used to predict potential target genes, which were integrated with the degradation group density file to identify common mRNAs as miRNA target genes. Finally, candidate target genes underwent Gene Ontology (GO) functional term and Kyoto Encyclopedia of Genes and Genomes (KEGG) annotation analysis using clusterProfiler v4.4.4 and KOBAS 3.0 software [60].

### 4.6. Validation of miRNA Target Genes by 5′-RLM-RACE

The RNA ligase-mediated RLM-5′ RACE experiments were performed to validate predicted target genes of low temperature-related miRNAs by using the GeneRaeer Kit (Invitrogen, Waltham, MA, USA) following the manufacturer′s instructions. Briefly, RNAs from both CK and low-temperature-treated Chinese jujube samples were ligated with an RNA adapter and reversely transcribed to cDNAs. Nested PCRs were carried out using the nested gene-specific primers (Table 7). The amplified PCR fragments were inserted into the pMD19-T vector for sequencing to determine the cleavage sites of target genes.

### 4.7. The Verification of Authenticity of High-Throughput Sequencing Data via qRT–PCR

To assess the reliability of the high-throughput sequencing data, quantitative reverse transcription polymerase chain reaction (qRT-PCR) was conducted on 7 miRNAs and 7 of their corresponding target genes associated with cold stress. Total RNA was extracted from the branches of *Ziziphus jujuba* Mill subjected to both control and cold treatment conditions. Reverse transcription was performed using the PrimeScript™ RT Reagent Kit (Takara, Dalian, China) in accordance with the manufacturer’s guidelines. Subsequently, real-time quantitative PCR analysis was executed with the Super Real PreMix Plus Kit (SYBR Green, TianGen, Beijing, China). 20 μL of PCR reaction contained 2 μL of 1:10 diluted cDNA, 1× SYBR Green Master Mix, and 0.2 μM of each forward and reverse primer; the relevant primers are shown in Table 8. All reactions were run on a Roche LightCycler 96 System (Roche, Basel, Switzerland). The cycling conditions were: 95 °C for 10 min, followed by 35 cycles of 95 °C for 15 s and 60 °C for 45 s. Each sample had triplicate biological replicates (independent plants) and three technical replicates (independent qPCR wells per biological replicate). Relative expression levels were determined using the 2^−ΔΔCt^ method. Expression levels were normalized to the internal control *St18sRNA* (for miRNAs) and ef1α (for target genes). Statistical significance between treatments was assessed using Student’s *t*-test (two-tailed) with ** *p* < 0.01 and * *p* < 0.05 considered significant. Error bars represent standard deviation (SD) from three biological replicates.

## 5. Conclusions

This study presents an exploratory analysis integrating small RNA sequencing and degradome sequencing to systematically identify candidate miRNAs and their target genes in *Ziziphus jujuba* Mill subjected to low-temperature stress. A total of 342 miRNAs were identified, with 177 demonstrating differential expression. These candidate miRNAs warrant further validation through prospectively designed experiments using intact plants, controlled temperature protocols, and functional assays to confirm their roles in freezing tolerance. Through degradome sequencing and functional annotation, nine miRNAs were identified as targeting 11 genes associated with the jujube tree’s response to low-temperature stress. Among these, the seven target sites of seven miRNAs were confirmed through RLM-5’RACE analysis. The verified miRNAs include cme-miR156a, gma-miR5368, novel_miR_100, novel_miR_139, novel_miR_181, novel_miR_41, and novel_miR_69, which target genes such as heat shock 70 kDa protein, low-temperature-induced cysteine proteinase-like, 2-methylene-furan-3-one reductase-like, bifunctional hydroxyethylthiazole kinase/thiamine-phosphate pyrophosphorylase, E3 ubiquitin-protein ligase RMA1H1-like isoform X2, glycine-rich RNA-binding protein RZ1A, 3-oxoacyl-[acyl-carrier-protein] reductase 4-like, 3-ketoacyl-CoA thiolase 2, peroxisoma, and heat shock cognate 70 kDa protein 2-like. This indicates that miRNAs and their target genes play a crucial role in the cold stress of *Ziziphus jujuba* Mill. This research not only establishes a crucial foundation for understanding the molecular regulatory mechanisms underlying the jujube tree’s adaptation to low temperatures but also identifies key targets for future molecular breeding efforts utilizing miRNAs. These resources may facilitate the development of molecular markers for cold resistance or the creation of new cold-resistant varieties via genetic engineering.

## Figures and Tables

**Table 1:** Statistical analysis of sequencing reads for the sRNAs libraries.

Samples	Raw Reads	Length < 18	Length > 30	Low Quality	Containing ’N’ Reads	Clean Reads	Q30 (%)
JCK-1	11,880,054	980,423	1,300,332	14	0	9,599,285	97.66
JCK-2	20,091,786	1,555,764	4,160,088	74	0	14,375,860	97.57
JCK-3	12,407,195	978,414	1,228,486	21	15	10,200,259	97.51
JT-1	11,984,901	1,629,827	408,846	11	11	9,946,206	96.94
JT-2	23,291,764	3,931,543	4,971,623	82	21	14,388,495	97.51
JT-3	25,731,472	5,438,588	612,127	10	0	19,680,747	97.14
LCK-1	18,879,492	3,275,289	656,252	25	49	14,947,877	97.67
LCK-2	18,806,959	3,289,705	681,630	14	0	14,835,610	97.56
LCK-3	19,328,728	2,908,853	707,600	14	0	15,712,261	97.82
LT-1	17,732,893	2,159,351	575,445	11	0	14,998,086	97.70
LT-2	21,449,008	3,709,847	781,846	19	9	16,957,287	96.08
LT-3	21,189,857	5,234,789	566,917	9	0	15,388,142	97.57

Note: JCK stands for ‘Jinchang No.1’ for comparison. JT is processed at Jinchang No.1. LCK corresponds to ‘Linzexiaozao’. LT is for processing ‘Linzexiaozao’.

**Table 2:** Comparison information statistics table with reference genome.

Sample ID	Total Reads	Mapped Reads	Mapped Reads (+)	Mapped Reads (−)
JCK-1	3,118,388	2,168,846 (69.55%)	1,558,267 (49.97%)	610,579 (19.58%)
JCK-2	4,888,628	3,439,727 (70.36%)	2,458,102 (50.28%)	981,625 (20.08%)
JCK-3	3,511,941	2,477,441 (70.54%)	1,770,309 (50.41%)	707,132 (20.14%)
JT-1	1,984,456	1,067,548 (53.80%)	717,668 (36.16%)	349,880 (17.63%)
JT-2	2,171,920	1,167,936 (53.77%)	851,887 (39.22%)	316,049 (14.55%)
JT-3	2,799,766	1,623,053 (57.97%)	1,183,171 (42.26%)	439,882 (15.71%)
LCK-1	2,354,770	1,576,954 (66.97%)	1,131,259 (48.04%)	445,695 (18.93%)
LCK-2	2,225,111	1,578,244 (70.93%)	1,132,278 (50.89%)	445,966 (20.04%)
LCK-3	5,247,506	3,795,281 (72.33%)	2,745,715 (52.32%)	1,049,566 (20.00%)
LT-1	4,197,273	2,942,576 (70.11%)	2,183,686 (52.03%)	758,890 (18.08%)
LT-2	4,929,712	3,261,659 (66.16%)	2,395,662 (48.60%)	865,997 (17.57%)
LT-3	4,471,410	3,059,486 (68.42%)	2,200,560 (49.21%)	858,926 (19.21%)

Note: JCK, JT, LCK, LT are the same as above.

**Table 3:** Statistical results of miRNA in each sample.

Sample ID	Known-miRNAs	Novel-miRNAs	Total
JCK-1	89	147	236
JCK-2	102	154	256
JCK-3	99	155	254
JT-1	85	106	191
JT-2	82	118	200
JT-3	82	122	204
LCK-1	72	181	253
LCK-2	73	182	255
LCK-3	94	158	252
LT-1	102	159	261
LT-2	100	168	268
LT-3	106	161	267
Total	123	219	342

**Table 4:** Statistical table of differentially expressed miRNAs.

DEG Set	DEG Number	Upregulated	Downregulated
JCK-1_JCK-2_JCK-3_vs_JT-1_JT-2_JT-3	112	61	51
LCK-2_LCK-3_LCK-1_vs_LT-1_LT-2_LT-3	102	22	80

**Table 5:** Summary of data from jujube tree degradome sequencing.

Sample	JC1 (Number)	JC1 (Ratio)	LZXZ (Number)	LZXZ (Ratio)
Raw Reads	9,847,384	/	10,188,488	/
Unique Raw Reads	4,464,727	/	4,352,961	/
reads < 15 nt after removing 3 adaptor	550,113	5.59%	321,026	3.15%
Mappable Reads	9,297,271	94.41%	9,867,462	96.85%
Unique reads < 15 nt after removing 3 adaptor	91,842	2.06%	72,571	1.67%
Unique Mappable Reads	4,372,885	97.94%	4,280,390	98.33%
Mapped Reads	4,529,612	46.00%	5,304,359	52.06%
Unique Mapped Reads	1,869,149	41.86%	1,798,804	41.32%
Number of input Transcript	50,542	/	50,542	/
Number of Covered Transcript	35,835	70.90%	34,895	69.04%

Note: JC1 (number) stands for ‘number of Reads in Jinchang No.1’; JC1 (ratio) stands for ‘ratio of component in raw Reads in Jinchang No.1’; LZXZ (number) stands for ‘number of Reads in Linzexiaozao’; LZXZ (ratio) stands for ‘ratio of component in raw Reads in Linzexiaozao’.

**Table 6:** Potential cold-related miRNAs and their target genes in Chinese jujube.

miRNAs	Target Genes ID	Targets Definition	Targets Annotation
cme-miR156a	LOC107423197	heat shock 70 kDa protein 8	GO:0006457 (protein folding); GO:0009408 (response to heat)
gma-miR5368	LOC107419994	low-temperature-induced cysteine proteinase-like	GO:0004197 (cysteine-type endopeptidase activity)
novel_miR_100	LOC107415384	2-methylene-furan-3-one reductase-like	GO:0009409 (response to cold)
	LOC107416875	myelin transcription factor 1-like protein	GO:0009409 (response to cold)
novel_miR_124	LOC107411166	two-component response regulator-like PRR73	GO:0009266 (response to temperature stimulus)
novel_miR_130	LOC107407137	Thiamine phosphate pyrophosphorylase (TMP-PPase family)	GO:0009409 (response to cold)
novel_miR_139	LOC107423986	E3 ubiquitin-protein ligase RMA1H1-like isoform X2	GO:0009409 (response to cold)
novel_miR_181	LOC107412024	glycine-rich RNA-binding protein RZ1A	GO:0009409 (response to cold); GO:0009631 (cold acclimation)
novel_miR_41	LOC107418159	3-oxoacyl-[acyl-carrier-protein] reductase 4-like	GO:0006633 (fatty acid biosynthetic process); ko00061(Fatty acid biosynthesis); ko01040 (Biosynthesis of unsaturated fatty acids)
	LOC107422570	3-ketoacyl-CoA thiolase 2, peroxisomal	GO:0006635 (fatty acid beta-oxidation); ko00071 (Fatty acid degradation); ko00592 (alpha-Linolenic acid metabolism); ko01040 (Biosynthesis of unsaturated fatty acids)
novel_miR_69	LOC107413527	heat shock cognate 70 kDa protein 2-like	GO:0009266 (response to temperature stimulus); GO:0009408 (response to heat)

**Table 7:** The nested PCR primers for the miRNA cleavage sites validation by RLM-5′ RACE.

Target Gene	Genomic Locust	Outer Primer 61	Inner Primer 70
heat shock 70 kDa protein 8	LOC107423197	GAATGTCACATATGACCGCATC	GCCATTCCAGACAGCAATGCTGC
low-temperature-induced cysteine proteinase-like	LOC107419994	CTGCTGCACAAATACCTTAGTGC	CAGTGCGCTTCAATGCCTTCACTCC
2-methylene-furan-3-one reductase-like	LOC107415384	CTGTAGCTGCCACTCTGGATG	CCAACTCCACCGGCACCACCTAG
myelin transcription factor 1-like protein	LOC107416875	CACAGTTCGCAGTTCTTCATCTC	GCAAGGTCTTAAGAGTCCGCTTGGAGTC
two-component response regulator-like PRR73	LOC107411166	CAAGAGAGTGCTACTTCCATTGTG	CATTGCTGAGCAGCAGCAGCCAAG
bifunctional hydroxyethylthiazole kinase/thiamine-phosphate pyrophosphorylase	LOC107407137	GCAGCAGCTAGCATCAGCTC	GCCAAATGCGCTGCCTTCTGTCC
E3 ubiquitin-protein ligase RMA1H1-like isoform X2	LOC107423986	CTAAGGAATTCTCCGAATTCAC	ACCAGGAATGGACTTCCATTCATGTG
glycine-rich RNA-binding protein RZ1A	LOC107412024	ACGACTGCCACCGTCTCTG	CGGTCAGGACCATAACGACCACCAC
3-oxoacyl-[acyl-carrier-protein] reductase 4-like	LOC107418159	CAAGAGCCAATGCAACAGC	GCTCCAGTGACGACAACCACAGGAG
3-ketoacyl-CoA thiolase 2, peroxisomal	LOC107422570	GGTCTTCTCTATCAACGCCTTC	GTGCTGCAACTATAACCACATCGTCG
heat shock cognate 70 kDa protein 2-like	LOC107413527	CGCAGAAGATGGAATCCAAC	CAGCACCACTACCACCAGCAGGAG

**Table 8:** Oligonucleotides used for qRT-PCR.

	Gene	Sequence of Oligonucleotides	Gene Length (bp)	TM (bp)
Primers for miRNAs	*cme-miR156a*	F 5′-UGACAGAAGAGAGUGAGCACGC-3′		60.5
	*gma-miR5368*	R 5’-GGACAGUCUCAGGUAGACAGC-3’		59.9
	*novel_miR_100*	F 5’-UAUUGGAUUUCUUUCUUUGCGC-3’		61.7
	*novel_miR_139*	F 5’-CGACUCCACCCAUUCCCA-3’		63.9
	*novel_miR_181*	F 5’-UUCCAUCUCUUGCACACUGC-3’		59.8
	*novel_miR_41*	F 5’-AAAGAGGAAGAGGGUGGUGC-3’		59.0
	*novel_miR_69*	F 5’-CAUCAUCUGUGCUCUCUAUCUC-3’		59.3
	*St18s RNA*	F 5’-TTAGAGGAAGGAGAAGTCGTAACAA-3’		59.8
Primers for target genes	LOC107423197	F 5’-ATGGAAGTCAGAATGCCTACAG -3’	194	58.3
		R 5’-CAATACCCAAGAACCACCAC -3’		57.8
	LOC107419994	F 5’-GCAAGGACAATCCATTAGGA -3’	172	57.6
		R 5’- AAGGAGAAACCAAGACCCTC -3’		57.3
	LOC107415384	F 5’-GAAAGAAGGTGGGAAATCG -3’	182	57.2
		R 5’- GCAAATGCCTCCAAAGTCT -3’		57.9
	LOC107423986	F 5’-GGAGAGATGGTTTATGCCAG -3’	170	57.2
		R 5’-GCACAGAAGAAAGCAGCAG -3’		57.4
	LOC107412024	F 5’-GATGACAAGCAAGCAATGG -3’	178	57.8
		R 5’-TTAGACCCACGACCAGAATC -3’		57.6
	LOC107418159	F 5’-GAATAACACGGGATGGGTT-3’	166	57.2
		R 5’-CACCAGACCAACAACAGATG-3’		57.5
	LOC107413527	F 5’-GCAAGAGCATCAATCCAGA-3’	150	57.4
		R 5’-ATAACACCTCCCGCAGTCT-3’		57.2
	ef1α	F 5’-ATTGGAAACGGATATGCTCCA-3’	101	61.6
		R 5’-TCCTTACCTGAACGCCTGTCA-3’		60.3

## Data Availability

The original contributions presented in this study are included in the article/Supplementary Materials. Further inquiries can be directed to the corresponding author.

## Supplementary Materials

The following supporting information can be downloaded at https://www.mdpi.com/article/10.3390/ijms27177912/s1.

## Abbreviation

cDNA	Complementary DNA
DEMs	Differentially Expressed miRNAs
FC	Fold Change
FDR	False Discovery Rate
GO	Gene Ontology
Hsp	Heat Shock Protein
KEGG	Kyoto Encyclopedia of Genes and Genomes
miRNA	MicroRNA
qRT-PCR	Quantitative Reverse Transcription Polymerase Chain Reaction
RLM-5′RACE	RNA Ligase-Mediated 5′ Rapid Amplification of cDNA Ends
sRNA	Small RNA
TPM	Transcripts Per Million
SPL	SQUAMOSA Promoter Binding Protein-Like
TCP	TEOSINTE BRANCHED1/CYCLOIDEA/PCF transcription factor
Hsp70	Heat Shock Protein 70
RZ1A	Glycine-rich RNA-binding Protein RZ1A
E3 ubiquitin ligase	E3 Ubiquitin-Protein Ligase
